# Xm^2^ Scores for Estimating Total Exposure to Multimodal Strategies Identified by Pharmacists for Managing Pain: Validity Testing and Clinical Relevance

**DOI:** 10.1155/2018/2530286

**Published:** 2018-12-12

**Authors:** David Rhys Axon, Sandipan Bhattacharjee, Terri L. Warholak, Marion K. Slack

**Affiliations:** Department of Pharmacy Practice and Science, The University of Arizona College of Pharmacy, 1295 N Martin Avenue, Tucson, AZ 85721, USA

## Abstract

**Objective:**

To assess the validity of an exposure score obtained from the Xm^2^ tool for all pharmacological and nonpharmacological strategies used by individuals to manage chronic pain.

**Methods:**

Using data from individuals with chronic pain, e**X**posure **m**ulti**m**odal (Xm^2^) scores were calculated by assigning one point for every 100 mg of morphine equivalent used (opioid medications); 25% of the maximum recommended exposure used (nonopioid medications); and any use of another strategy then summed. Content, criterion, construct, and convergent validity were assessed.

**Results:**

The sample of 149 individuals used a mean of 12.6 (SD = 4.6) strategies to manage pain and had a mean Xm^2^ score of 16.8 (SD = 9.1). Content validity was established by demonstrating that the pain management strategies identified were also reported in the literature. Criterion validity was established by the positive association of exposure scores with the following: interference with work (odds ratio (OR) = 2.23, 95% confidence interval (CI) = 1.14–4.36), daily activities (OR = 2.10, CI = 1.07–4.13), relationships (OR = 1.98, CI = 1.01–3.88), and leisure activities (OR = 2.31, CI = 1.18–4.50); workdays missed (OR = 5.10, CI = 1.92–13.58); emergency department visits (OR = 3.40, CI = 1.17–9.91); hospitalizations (OR = 4.18, CI = 0.86–20.37); and by a negative association with satisfaction (OR = 0.40, CI = 0.18–0.88). Construct validity was established by the positive association of exposure with baseline pain intensity (*p* < 0.01) and odds of experiencing an adverse event (OR = 2.31, CI = 1.18–4.52). Convergent validity was established through correlations of pain intensity from the Xm^2^ score and existing quantitative analgesic questionnaire (QAQ) score.

**Discussion:**

Xm^2^ scores represent a valid estimate of total exposure to multimodal strategies used and provide clinically relevant information for deciding what strategies to use at what level.

## 1. Introduction

Chronic pain is defined by the American Chronic Pain Association as “ongoing or recurrent pain, lasting beyond the usual course of acute illness or injury or more than three to six months, and which adversely affects the individual's well being” [1]. Chronic pain affected approximately 25 million adults in the United States (US) in 2012 [2]. Pain is a major cause of disability and results in frequent physician visits, the need to take medication, and poorer quality of life [3]. The total cost of pain to society ranged from $560 to $635 billion (2010 US dollars) per year in the US [3, 4]. Appropriate pain relief is considered a basic human right, and individuals experiencing pain should expect to receive adequate pain management [5]. However, adequate management is dependent on the total dose or exposure to all medications and other strategies used for managing pain [3, 6]. That is, managing the concentration or intensity, duration, and frequency of the exposure [7].

Population-based surveys of community dwelling adults with chronic pain have shown that a wide variety of strategies are used to manage pain. For example, strategies include analgesic medications such as opioids and nonsteroidal anti-inflammatory drugs (NSAIDs), medications such as anticonvulsants and antidepressants, and nonpharmacological strategies such as exercise, hot and cold modalities, massage, and using dietary supplements among many others [6, 8–24]. Despite the variety of strategies used by individuals with pain, Breivik et al. [12] found 40% to 64% of respondents indicated that their pain management was unsatisfactory. However, these surveys do not report doses of medications used or exposure level to other management strategies. Given that the effects of the medications and other strategies are dependent on an adequate exposure to the treatment strategy being used, it would be helpful to be able to quantify actual exposure. If actual exposure is not resulting in a therapeutic response, then exposure can be adjusted up to the maximum recommended exposure (MRE) before adding or changing treatment strategies. Despite the need for exposure information, a method for combining dose or exposure data from pharmacological and nonpharmacological strategies is not currently available.

A method for standardizing and combining pharmacological doses is available. The Quantitative Analgesic Questionnaire (QAQ) was developed by Robinson-Papp et al. [25] to assess adherence to pain medications by standardizing doses based on percent of the maximum recommended dose used or for opioids and standardizing doses based on morphine milligram equivalents (MMEs) calculated from the doses that patients say they actually use. Actual dose used can then be compared to recommended dose. The QAQ is limited though in that it does not include medications that are not typically used for pain or any nonpharmacological strategies.

In this study, we report an extension of the QAQ tool to include nonpharmacological strategies, including medical (e.g., physician visits), physical (e.g., exercise), psychological (e.g., distraction), and self-initiated strategies (e.g., change position) to obtain a total exposure score (e**X**posure **m**ulti**m**odal, i.e., the Xm^2^ score) for all strategies used. Such a score allows clinicians and researchers to relate the total exposure of all strategies to outcomes, that is, to relate the use of both pharmacological and nonpharmacological strategies to pain intensity or to reduction of interference with daily activities. If the Xm^2^ score is valid, then users can have confidence that the scores from the Xm^2^ tool represent total exposure to strategies used by an individual to manage pain. The purpose of this study was to (1) assess the validity of the Xm^2^ score estimated for all strategies (pharmacological and nonpharmacological) used by individuals to manage chronic pain and (2) discuss the clinical relevance of the Xm^2^ scores.

## 2. Methods

### 2.1. Data Source

Data for testing the validity of the Xm^2^ score were obtained from a previous study [26] completed by pharmacists who personally had chronic pain and who were licensed in a southwestern state in the US. Pharmacists were surveyed because they could provide detailed information on their management strategies, including doses of medications [26].

The survey collected data on the types and doses of prescription and over-the-counter (OTC) medications used. Data were also collected on medical strategies (e.g., surgery), physical strategies (e.g., exercise), psychological strategies (e.g., distraction), and self-initiated strategies (e.g., home remedies) used; however, no data were obtained on the exposure (dose) of the nonpharmacological strategies used. Data also were collected on outcomes, including pain intensity after treatment, interference with work, daily activities, relationships, satisfaction with pain management strategies, workdays lost, emergency department visits, adverse events, and hospitalizations. Additionally, data on pain (e.g., baseline pain levels) and demographic characteristics were also collected. Further details are reported elsewhere [26].

### 2.2. Exposure Scoring Procedure

A total exposure score was calculated for each individual by adapting the methods described by Robinson-Papp et al. [25], whereby points were assigned according to the exposure (dose) of each medication used and summed. Points for opioid medications were obtained by converting the dose to a weekly morphine milligram equivalent (MME) and scored one point for every 100 milligrams (mg) of morphine equivalent used per week (i.e., 1–99 mg = one point, 100–199 mg = two points, etc.). For example, a score of 5 would indicate that an individual was using between 400 MMEs and 499 MMEs of opioid per week or 57–71 MMEs per day. Given that 57 MMEs per day approximates the CDC's recommended safe dose of 50 MMEs per day [27], we used 400 MMEs per week as a benchmark for interpreting Xm^2^ scores for opioids. For nonopioid prescription medications, one point was assigned for every 25 percent of the maximum recommended exposure (MRE) used (i.e., 1–24% = one point, 25–49% = two points, etc.). For example, a score of 5 would indicate that a specific nonopioid medication was being used at 100 to 124% of the maximum recommended exposure. Because there were no data on exposure (dose) for nonmedication strategies, we assigned one point for each nonmedication strategy used, including medical, physical, psychological, and self-initiated strategies.

### 2.3. Validity Assessment

The first step in the validity assessment was to describe the characteristics of the Xm^2^ scores. The score characteristics included the number and range of the scores, as well as the categories of pain management identified. Interpretation of the scores is illustrated so that validity can be considered in the context of the information that the scores provide. The validity of the Xm^2^ tool was assessed by investigating the following: (1) content validity; (2) criterion validity; (3) construct validity; and (4) convergent validity.

#### 2.3.1. Content Validity

Content validity has been defined as indicating if the sample of the target behaviors is representative of the behaviors in the domain [28]. For this study, content validity was assessed by examining the questionnaire, data, and source population. Evidence of content validity was present if (1) the types of pain management strategies used for calculation of the Xm^2^ score were representative of those used by individuals, (2) the questionnaire to collect data on types (and doses) of self-reported pain management strategies represented known strategies (i.e., identified in the literature), and (3) the source population had knowledge and experience with personal pain management and were able to provide that information via an online questionnaire.

#### 2.3.2. Criterion Validity

Criterion validity can be defined as the association of a measure with outcomes [28]. Evidence of criterion validity was present if exposure scores were associated with outcomes. We hypothesized that higher exposure scores would be associated with more interference with daily activities, leisure activities, work, relationships, workdays lost, use of emergency departments, and hospital admissions. In contrast, higher exposure scores would be negatively related to satisfaction with pain management.

#### 2.3.3. Construct Validity

Construct validity represents the theory or the premise on which the score is based [28]. Evidence of construct validity was present if (1) higher exposures were associated with higher levels of pain and (2) exposure to greater numbers of strategies was associated with a higher risk of adverse events. A multivariable regression was conducted to explore whether the exposure score was independently related to baseline pain and adverse events.

#### 2.3.4. Convergent Validity

Convergent validity compares the measure under investigation to other measures of the same construct [28]. Evidence of convergent validity was present if the correlation of the Xm^2^ scores to baseline pain were comparable to the correlation of the QAQ score with baseline pain [25].

### 2.4. Data Analysis

A median split was used to categorize exposure scores as low (below the median exposure score of 16) or high (equal to or greater than 16). Demographic characteristics and outcomes were compared between the low- and high-exposure groups using chi-square or Fisher's exact tests for nominal data and *t*-tests for normally distributed continuous data. To describe the distributions of the exposure scores and the number of pain management strategies used, a histogram was constructed to display Xm^2^ scores and number of strategies used versus the percent of individuals with each score or number of strategies reported. Findings related to construct and criterion validity were reported as odds ratios with 95% confidence intervals [29].

The independence of the association between exposure score and baseline pain was tested using multivariable linear regression, and the independent association of exposure score to adverse events was tested using multivariable logistic regression. In both models, the key independent variable was exposure score, and characteristics of the individuals were used as control variables. All covariates were included in the linear model, while age, disability, and employment status were included in the logistic model (identified using the backward elimination technique). The assumptions of the models were tested. An alpha value of 0.05 selected a priori was used to determine significance. Analyses were performed using SAS software version 9.4 (SAS Institute Inc., Cary, NC, USA).

## 3. Results

### 3.1. Demographic Characteristics

Of the 149 respondents, 73 (49%) were in the low-exposure group and 76 (51%) in the high-exposure group. Compared to the low-exposure group, the high-exposure group reported significantly higher baseline pain levels (mean = 7.5 ± 1.9 vs. mean = 6.2 ± 2.1; *p* < 0.01), more individuals with a disability (18.7% vs. 2.8%; *p* < 0.01), and more individuals who had completed continuing education in pain management (93.2% vs. 81.9%; *p*=0.04). Individuals in the higher exposure group had significantly more emergency department visits in the last five years due to pain (*p*=0.02), more adverse events from pain management strategies (*p*=0.01), more pain interference on daily activities (*p*=0.03), more pain interference on leisure activities (*p*=0.01), more pain interference on relationships (*p* < 0.05), more pain interference on work (*p*=0.02), and more workdays lost in the last six months due to pain (*p* < 0.01) and were significantly less satisfied with their pain management strategies than those in the lower exposure group (67.1% vs. 83.6%, respectively, *p*=0.02) (Table 1).

### 3.2. Characteristics of the Xm^2^ Scores

As shown in Table 2, Xm^2^ scores were available for 99 different management strategies that represented 15 categories of medications and four nonpharmacological categories (medical strategies, physical strategies, psychological strategies, and self-initiated strategies). The Xm^2^ scores ranged from 1 to 15 for individual pharmacological strategies. Scores for nonpharmacological strategies were 1 for all individual strategies because there were no data on exposure level.

The characteristics of the exposure levels, represented by the Xm^2^ scores, also are shown in Table 2. The exposure levels were generally moderate for both opioid and nonopioid medications. The average Xm^2^ score for opioid use was 3.2 (SD = 4.1; range = 1–15) representing about 80% of the benchmark exposure of 400 MMEs per week or about 57 MMEs per day. However, the highest Xm^2^ score was 15, which represents 3.75 times the benchmark of 400 MMEs per week. The average Xm^2^ score for nonopioid medications was 2.6 (SD = 3.1), 52% of the MRE (Xm^2^ score of 5). The two nonopioid medications with the highest Xm^2^ scores were duloxetine (maximum Xm^2^ score = 9) and cyclobenzaprine (maximum Xm^2^ score = 9); hence they were used at exposures 1.8 times the recommended exposure.

**Figure 1:**
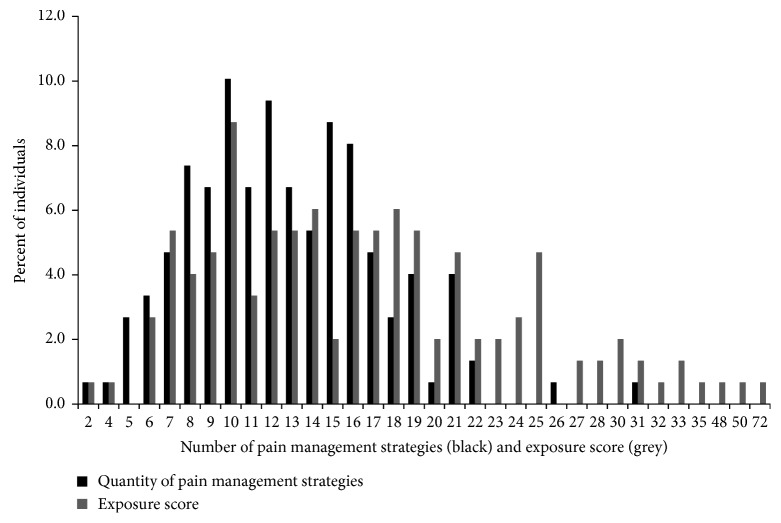
Percent of individuals using each number of pain self-management strategies and percent of individuals assigned each exposure score. The black bars indicate the number of pain management strategies used. The grey bars indicate the exposure score. For example, Figure 1 shows 10.1% of individuals used 10 pain management strategies and 8.7% of individuals had an exposure score of 10, while 0.7% of individuals used 20 pain management strategies and 2% of individuals had an exposure score of 20. Mean number of strategies used = 12.6 (standard deviation = 4.6). Mean exposure score = 16.8 (standard deviation = 9.1).

The range of the number of management strategies used and the range of Xm^2^ scores are shown graphically in Figure 1. The Xm^2^ scores for individual respondents (grey bars) ranged from 2 to 72 and had a distribution skewed to the right. The mode was 10, the median 16, and the mean 16.8 (SD = 9.1); the coefficient of variation was 54%. The number of pain management strategies used by individuals (black bars) ranged from 2 to 31 with a normal distribution. The mode was 12, the median 12, and the mean 12.6 (SD = 4.6); the coefficient of variation was 37%.

### 3.3. Evidence of Content Validity

The questionnaire required respondents to report specific categories of prescription therapies used and then identify the specific medication and the total daily dose. A similar strategy was used for OTC medications and nonpharmacological strategies, except respondents did not provide dose data. An “other” category allowed respondents to add strategies not specifically identified in the questionnaire. Hence, the questionnaire was able to collect data on all pain management strategies recalled at the time of completion.

In general, the strategies included were identifiable in the literature. A total of 99 different strategies were identified, including 13 different classes of pharmacological strategies plus OTC products and “other.” Examples of nonpharmacological strategies included physical activity, acupuncture, massage, meditation, relaxation, avoiding specific activities, changing body position, diet modifications, transcutaneous electrical nerve stimulation (TENS), hot/cold packs, and rest (Table 2). Sixty-one percent (60 of 99) of the strategies identified in this survey and 48% (31 of 65) of the prescription medications represented classes of medications were present in the literature [6, 8–24, 30–34].

Survey respondents were pharmacists who were highly trained in medication management describing their personal pain management strategies and who had completed continuing education on pain management (87.7%). Therefore, there is evidence that the source population had the ability to provide information on both types and exposure to pain management strategies. Overall, the evidence is more than adequate for content validity.

### 3.4. Evidence of Criterion Validity

**Figure 2:**
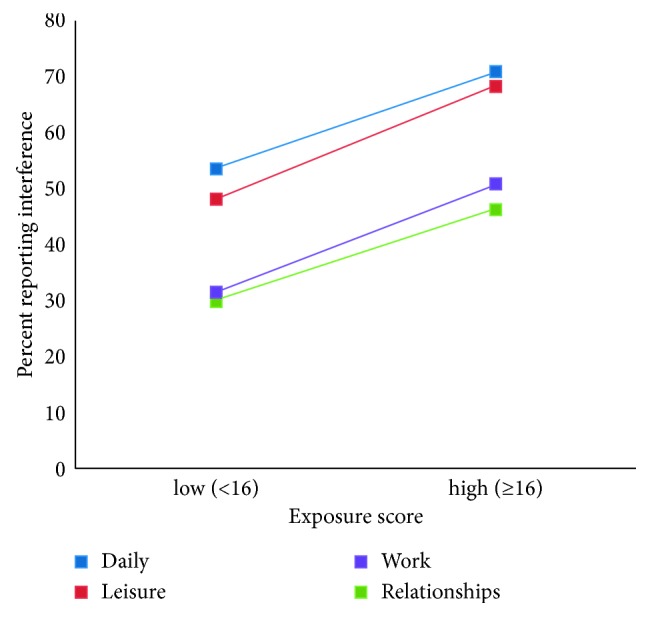
Percent of individuals reporting interference in the low- or high-exposure score groups. Daily = interference on daily activities: OR = 2.10 (95% CI = 1.07–4.13). Leisure = interference on leisure activities: OR = 2.31 (95% CI = 1.18–4.50). Work = interference on work: OR = 2.23 (95% CI = 1.14–4.36). Relationships = interference on relationships: OR = 1.98 (95% CI = 1.01–3.88). For example, Figure 2 shows that 53% of individuals in the low-exposure group and 71% of individuals in the high-exposure group reported that pain interfered with their daily activities.

**Figure 3:**
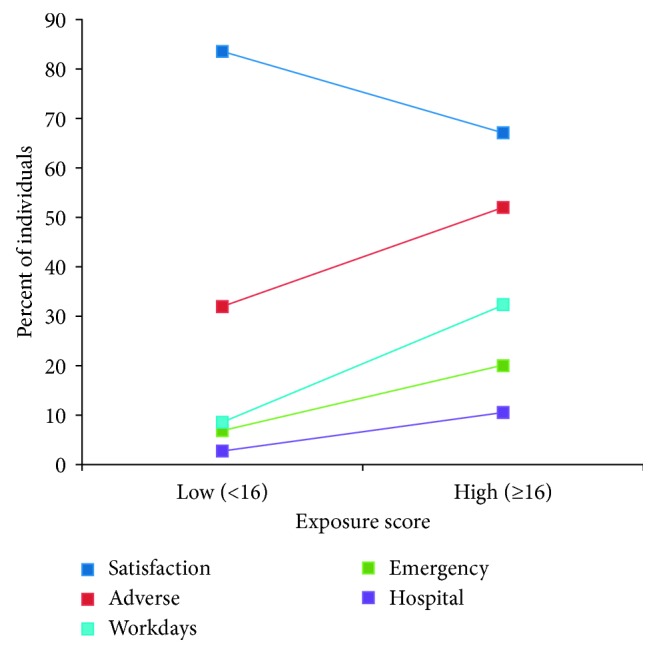
Percent of individuals reporting pain outcomes in the low- or high-exposure score groups. Satisfaction = satisfaction with pain management strategies: OR = 0.40 (95% CI = 0.18–0.88). Adverse = adverse events from pain management strategies: OR = 2.31 (95% CI = 1.18–4.52). Workdays = workdays lost due to pain in past six months: OR = 5.10 (1.92–13.58). Emergency = emergency department visits due to pain in past five years: OR = 3.40 (95% CI = 1.17–9.91). Hospital = hospital admissions due to pain in past five years: OR = 4.18 (95% CI = 0.86–20.37). For example, Figure 3 shows that 32% of individuals in the low-exposure group and 52% of individuals in the high-exposure group reported that they had experienced an adverse event due to their pain management strategies.

There is also substantial evidence that the exposure score is related to pain outcomes as is required for criterion validity. As shown in Table 1 and Figures 2 and 3, the association of the exposure score was positive for all outcomes except satisfaction, which had a negative association as expected.

### 3.5. Evidence of Construct Validity

Assessment of the purported association between the Xm^2^ scores and baseline pain and adverse events both provided evidence for construct validity. Baseline pain was positively related to exposure; the higher the level of baseline pain, the higher the exposure score (*p* < 0.01; see Table 1). That is, the higher the level of baseline pain, the more management strategies with higher exposures were used to manage the pain. A similar positive association was found for adverse events; the higher the exposure score, the greater the proportion of respondents who had experienced an adverse event (OR = 2.31, 95% CI = 1.18–4.52; see Figure 3).

The multivariable linear regression model (adjusted for age, sex, race, disability status, health status, marital status, employment status, years practiced, professional practice site, and completed continuing education on pain) for exposure score on baseline pain indicated that for every one unit increase in exposure score, mean baseline pain increased by 0.07 (standard error 0.03, *p*=0.01, *r*^2^=0.22). The regression also indicated that exposure is an independent predictor of baseline pain and hence is not an artifact of an associated variable. The multivariable logistic regression model (adjusted for age, employment status, and disability) for exposure score on adverse events indicated that for every one unit increase in exposure score, the odds of having experienced an adverse event increased by eight percent (OR = 1.08, 95% CI = 1.07–1.15, *p*=0.01, c-statistic = 0.79). Exposure was also an independent predictor of adverse events.

### 3.6. Evidence of Convergent Validity

Evidence of convergent validity was available from a comparison of the correlation between Xm^2^ and the QAQ scores and patient-reported baseline pain intensity. Our adjusted multivariable linear regression (described above) indicated an increase in baseline pain as exposure score increased, and Robinson-Papp et al. report that their QAQ score was correlated with patient-reported pain intensity (*r*=0.38, *p* < 0.001).

## 4. Discussion

To the best of our knowledge, this is the first study to show that an overall score indicating level of exposure to all pain management strategies, pharmacological and nonpharmacological, can provide valid information and has a clinically meaningful association with baseline pain and outcomes such as interference, emergency department use, and hospitalizations. Because the Xm^2^ score is based on quartiles of the recommended total exposure, it represents ratio level measurement with a meaningful zero and equal intervals for nonopioid management strategies. Hence, the score is standardized and is comparable regardless of the specific strategy being considered or the level of the recommended maximum exposure.

A score that is valid can provide a variety of clinically relevant information. First, the Xm^2^ score is informative because it describes what individuals are actually doing to manage their pain, and individuals in this sample were clearly using multimodal strategies. The average individual used 12.6 different strategies and had an average Xm^2^ score of 16.8. It appears that by using more strategies at a higher exposure level, individuals with higher levels of pain were able to achieve posttreatment levels of pain similar to the lower pain group. Second, the Xm^2^ score indicates the level at which each strategy is being used; hence, it is possible to identify whether specific strategies are being overused or underused.

The Xm^2^ score also enables the clinician to obtain an estimate of risk for adverse events and interference in activities. Individuals with Xm^2^ scores at or above the median (16) were more than two times more likely to experience an adverse event or interference with work, daily activities, leisure activities, and relationships than the lower exposure group even though there were no differences in posttreatment pain intensity.

Our findings also showed that pharmacists in this study were knowledgeable about different pain management strategies. Given that pharmacists are highly trained and accessible healthcare professionals, health systems should consider expanded roles for pharmacists in helping individuals in the general population manage their chronic pain. Use of the Xm^2^ score, in collaboration with healthcare professionals such as pharmacists or physicians, may help individuals manage their pain more effectively and consequently improve their quality of life.

Robinson-Papp et al. [25] initially developed the scoring system used here to assess adherence that also provides useful information to clinicians. We have shown that the scoring system can be expanded to include nonpharmacological strategies for managing pain and that the scores provide information beyond adherence to enable clinicians to improve decision making related to the quantity and exposure to each strategy used for managing pain.

Although the Xm^2^ scores were shown to be valid and provide useful information, they are limited in that there were no data available on exposure level for nonpharmacological strategies. Each nonpharmacological strategy was scored 1 that represents up to 24% of the MRE. Hence, nonpharmacological strategies contributed limited information to the Xm^2^ score. Part of the issue for nonpharmacological strategies is defining the MRE. There is currently an attempt to establish a recommended exposure for chiropractic care, specifically an approach known as spinal manipulation [35]. Perhaps that effort will provide a model for establishing recommended exposure for other nonpharmacological strategies in future.

Additionally, more work is needed to assure the scoring for opioids is consistent with nonopioids. Robinson-Papp et al. [25] did not relate opioid exposure to a MRE so we adapted the approach by using 400 MMEs (57 MMEs) to represent a benchmark for interpretation of scores for opioids. There does not appear to be consensus on the MRE for opioids although the CDC has produced some guidance [27], however, further efforts perhaps involving a Delphi panel are needed.

### 4.1. Implications for Future Research

Based on the findings from this study, we recommend that future research into pain management from a patient perspective includes a measure of treatment burden. Individuals in this study were using up to 31 different strategies (average 12.6) to manage their pain, which represents a substantial treatment burden. Indeed the large number of strategies used by the higher exposure group may be reflected in the higher ratings for pain interference and with the reduced satisfaction with their pain management.

More studies are needed on self-reported pain management strategies. Studies using clinical data (e.g., chart reviews) are limited in that nonpharmacological strategies are not likely to be identified. Also, this study did not provide information on pain management strategies used by the general population; this was a group with expertise in pain management that may have resulted in their using more strategies than the average person. Given that they appeared to be able to reduce their pain on average to tolerable levels using a large number of strategies to manage their pain, further investigation into the use of multiple strategies seems worthwhile.

A third area for additional research is related to the development of an Xm^2^ tool that could be used by the general population. A website or an “app” that assists patients with reviewing their pain management strategies should be useful. For example, an individual could identify that they are not using nonpharmacological strategies such as exercise, psychological strategies, or even simple self-initiated strategies such as changing position and could add them to their pain management plan. Further, it may increase individuals' ability to understand that they need to use management strategies at a specific level to benefit from them that may increase adherence.

### 4.2. Limitations

There were several limitations to this study. The sample size was relatively small and not representative of the general population. The sample also consisted of licensed pharmacists; individuals with more extreme pain may no longer be working or licensed, thus truncating our findings. However, for testing a new tool, it was beneficial to have a sample with expertise as individuals needed to provide the exposure information via an online questionnaire that would be difficult for the general population. Use of a questionnaire also introduces the likelihood of response bias and is limited by subjects' inability to recall accurately.

### 4.3. Conclusion

Initial testing of the Xm^2^ tool indicates that Xm^2^ scores represent a valid estimate of total exposure to multimodal strategies and that they can provide clinically relevant information to inform decisions concerning which strategies to use at what level for managing chronic pain.

## Figures and Tables

**Table 1:** Demographic characteristics and pain outcomes of study participants.

Characteristic	Total (*n* = 149)	Xm^2^ score (points)		*p* value
		Low (<16) (*n* = 73)	High (≥16) (*n* = 76)	
Baseline pain (*n* = 121) mean (standard deviation)	6.9 (2.1)	6.2 (2.1)	7.5 (1.9)	<0.01
Age (*n* = 146) mean (standard deviation)	52.5 (13.9)	52.5 (13.8)	52.4 (14.1)	0.94
Sex, female (*n* = 147) *N* (%)	77 (52.4)	36 (50.0)	41 (54.7)	0.57
Race, white (*n* = 146) *N* (%)	131 (89.7)	64 (90.1)	67 (89.3)	0.87
Disability (*n* = 147) *N* (%)	16 (10.9)	2 (2.8)	14 (18.7)	<0.01
Health status (*n* = 148) *N* (%)				
Poor/fair	26 (17.6)	10 (13.9)	16 (21.1)	0.25
Good/excellent	122 (82.4)	62 (86.1)	60 (79.0)	
Marital status, married (*n* = 143) *N* (%)	110 (76.9)	56 (81.2)	54 (73.0)	0.25
Employment status, employed (*n* = 148) *N* (%)	124 (83.8)	58 (80.6)	66 (86.8)	0.30
Primary professional practice site (*n* = 147) *N* (%)				
Community	58 (39.5)	24 (33.3)	34 (45.3)	0.26
Hospital	25 (17.0)	12 (16.7)	13 (17.3)	
Other	64 (43.5)	36 (50.0)	28 (37.3)	
Completed continuing education on pain management (*n* = 146) *N* (%)	128 (87.7)	59 (81.9)	69 (93.2)	0.04
Years practiced (*n* = 146) mean (standard deviation)	24.8 (14.5)	25.1 (15.0)	24.4 (14.1)	0.77
Hospital admissions in last five years due to pain (*n* = 149) *N* (%)	10 (6.7)	2 (2.7)	8 (10.5)	0.10
Emergency department visits in last five years due to pain (*n* = 148) *N* (%)	20 (13.5)	5 (6.9)	15 (20.0)	0.02
Adverse events from any strategies used (*n* = 147) *N* (%)	62 (42.2)	23 (31.9)	39 (52.0)	0.01
Pain interference on daily activities (*n* = 148) *N* (%)	92 (62.2)	39 (53.4)	53 (70.7)	0.03
Pain interference on leisure activities (*n* = 148) *N* (%)	86 (58.1)	35 (48.0)	51 (68.0)	0.01
Pain interference on relationships (*n* = 149) *N* (%)	57 (38.3)	22 (30.1)	35 (46.1)	<0.05
Pain interference on work (*n* = 148) *N* (%)	61 (41.2)	23 (31.5)	38 (50.7)	0.02
Satisfaction with pain management strategies (*n* = 149) *N* (%)	112 (75.2)	61 (83.6)	51 (67.1)	0.02
Workdays lost in last six months due to pain (*n* = 138) *N* (%)	28 (20.3)	6 (8.6)	22 (32.4)	<0.01
Pain intensity after treatment (*n* = 136) mean (standard deviation)	3.2 (2.2)	3.0 (2.3)	3.3 (2.1)	0.54
Percent pain relief from all strategies (*n* = 149) mean (standard deviation)	69.1 (20.4)	70.8 (20.8)	67.4 (20.1)	0.32

All comparisons conducted between low- and high-exposure groups using *t*-test for baseline pain, age, and years practiced and chi-squared test for all remaining variables.

**Table 2:** Number of individuals using each pain management strategy identified in the study organized by low or high Xm^2^ score and maximum Xm^2^ score for each strategy.

Pain management strategy	Individuals using each strategy N (%)			Maximum Xm^2^ score
	Total (*n* = 149)	Low Xm^2^ score (<16 points) (*n* = 73)	High Xm^2^ score (≥16 points) (*n* = 76)	
Prescription medications				
Use of an opioid^*∗*^	49 (32.9)	11	38	15
Codeine^*∗*^	3 (2.0)	1	2	2
Fentanyl^*∗*^	1 (0.7)	0	1	1
Hydrocodone^*∗*^	26 (17.4)	5	21	6
Hydromorphone	1 (0.7)	0	1	7
Methadone	1 (0.7)	0	1	13
Morphine^*∗*^	2 (1.3)	0	2	13
Oxycodone^*∗*^	13 (8.7)	2	11	15
Tramadol^*∗*^	11 (7.4)	3	8	3
Use of a nonopioid	125 (83.9)	51	74	9
Analgesics^*∗*^				
Acetaminophen^*∗*^	36 (24.2)	6	30	3
Dichloralphenazone	1 (0.7)	0	1	2
Anticonvulsants^*∗*^				
Carbamazepine^*∗*^	1 (0.7)	1	0	3
Gabapentin^*∗*^	17 (11.4)	5	12	4
Lamotrigine^*∗*^	1 (0.7)	0	1	2
Levetiracetam^*∗*^	1 (0.7)	0	1	3
Pregabalin	2 (1.3)	2	0	2
Topiramate	3 (2.0)	0	3	2
Valproic acid	1 (0.7)	0	1	3
Antidepressants^*∗*^				
Amitriptyline^*∗*^	8 (5.4)	2	6	7
Bupropion	1 (0.7)	0	1	2
Duloxetine^*∗*^	12 (8.1)	1	11	9
Fluoxetine^*∗*^	1 (0.7)	0	1	3
Mirtazapine	1 (0.7)	0	1	2
Nortriptyline	4 (2.7)	0	4	4
Sertraline	2 (1.3)	0	2	3
Trazodone	2 (1.3)	0	2	2
Venlafaxine^*∗*^	4 (2.7)	0	4	6
Vilazodone	1 (0.7)	0	1	5
Antipsychotic				
Quetiapine	1 (0.7)	0	1	1
Barbiturate^*∗*^				
Butalbital^*∗*^	3 (2.0)	0	3	5
Beta blockers^*∗*^				
Atenolol	1 (0.7)	0	1	3
Metoprolol	3 (2.0)	0	3	2
Propanolol	3 (2.0)	0	3	4
Calcium channel blockers^*∗*^				
Amlodipine	1 (0.7)	0	1	5
Verapamil	1 (0.7)	0	1	3
Muscle relaxants^*∗*^				
Baclofen	1 (0.7)	0	1	2
Carisoprodol	9 (6.0)	1	8	5
Chlorzoxazone	1 (0.7)	0	1	2
Cyclobenzaprine^*∗*^	29 (19.5)	5	24	9
Metaxalone	4 (2.7)	0	4	4
Methocarbamol^*∗*^	6 (4.0)	1	5	2
Orphenadrine	1 (0.7)	0	1	3
Tizanidine	3 (2.0)	0	3	1
Nonsteroidal anti-inflammatory drugs^*∗*^				
Celecoxib^*∗*^	9 (6.0)	0	9	5
Diclofenac^*∗*^	7 (4.7)	1	6	5
Etodolac^*∗*^	2 (1.3)	1	1	5
Ibuprofen^*∗*^	58 (38.9)	27	31	4
Indomethacin^*∗*^	2 (1.3)	0	2	2
Meloxicam^*∗*^	12 (8.1)	1	11	5
Nabumetone	4 (2.7)	1	3	4
Naproxen^*∗*^	37 (24.8)	13	24	4
Salsalate	2 (1.3)	0	2	2
Sulindac	1 (0.7)	1	0	3
Tolmetin	1 (0.7)	0	1	3
Sedatives				
Alprazolam^*∗*^	1 (0.7)	0	1	1
Diazepam^*∗*^	1 (0.7)	0	1	3
Lorazepam^*∗*^	2 (1.3)	0	2	1
Steroids^*∗*^				
Methylprednisolone^*∗*^	1 (0.7)	0	1	1
Prednisolone^*∗*^	1 (0.7)	0	1	1
Triptans^*∗*^				
Eletriptan	3 (2.0)	0	3	3
Naratriptan	1 (0.7)	0	1	3
Rizatriptan	4 (2.7)	1	3	2
Sumatriptan^*∗*^	5 (3.4)	0	5	3
Zolmitriptan	2 (1.3)	1	1	3
Others				
Caffeine	3 (2.0)	0	3	1
Isometheptene	1 (0.7)	0	1	2
Nonprescription medications^*∗*^				
Acetaminophen^*∗*^	64 (43.0)	28	36	1
Aspirin^*∗*^	16 (10.7)	8	8	1
Benadryl	1 (0.7)	0	1	1
Excedrin^*∗*^	2 (1.3)	1	1	1
Famotidine	1 (0.7)	0	1	1
Herbals^*∗*^	16 (10.7)	7	9	1
NSAIDs^*∗*^	95 (63.8)	48	47	1
Supplement^*∗*^	11 (7.4)	3	8	1
Medical strategies				
Chiropractor^*∗*^	32 (21.5)	12	20	1
Nasal spray	1 (0.7)	0	1	1
Other injections^*∗*^	1 (0.7)	0	1	1
Patch^*∗*^	2 (1.3)	0	2	1
Surgery^*∗*^	31 (20.8)	5	26	1
Steroid injections	50 (33.6)	10	40	1
Topical products^*∗*^	15 (10.1)	5	10	1
Physical strategies				
Acupuncture^*∗*^	21 (14.1)	6	15	1
Massage^*∗*^	92 (61.7)	39	53	1
Physical activity^*∗*^	105 (70.5)	44	61	1
Physical therapy^*∗*^	81 (54.4)	27	54	1
Stretching^*∗*^	5 (3.4)	5	0	1
TENS^*∗*^	51 (34.2)	14	37	1
Psychological strategies				
Meditation^*∗*^	42 (28.2)	12	30	1
Relaxation^*∗*^	89 (59.7)	31	58	1
Self-initiated strategies				
Alcohol^*∗*^	1 (0.7)	1	0	1
Avoid specific activities^*∗*^	104 (69.8)	43	61	1
Cannabis/Heroin^*∗*^	3 (2.0)	0	3	1
Changing body position^*∗*^	132 (88.6)	61	71	1
Diet^*∗*^	3 (2.0)	2	1	1
Education	38 (25.5)	12	26	1
Essential oils^*∗*^	1 (0.7)	0	1	1
Hot bath or shower^*∗*^	96 (64.4)	39	57	1
Hot or cold packs^*∗*^	99 (66.4)	36	63	1
Rest^*∗*^	125 (83.9)	56	69	1
Unspecified^*∗*^	59 (39.6)	27	32	1

TENS = transcutaneous electrical nerve stimulation. The minimum score is 0 in all cases. ^*∗*^Indicates the strategy was also identified in the literature. An Xm^2^ score of 5 indicates that item is being used at 100 to 124% of the recommended maximum exposure (RME).

## Data Availability

The survey data used to support the findings of this study are available from the corresponding author upon request.
